# Challenging Management of a Breast Mass: Case Report and Literature Review

**DOI:** 10.7759/cureus.41855

**Published:** 2023-07-13

**Authors:** Saleh Alhalaseh, Jordan Smith, Nmair Alziadin, Liliya Gandrabur

**Affiliations:** 1 Internal Medicine, St. Barnabas Hospital Health System, Bronx, USA; 2 Cardiovascular Medicine, University of Nebraska Medical Center, Omaha, USA; 3 Rheumatology, St. Barnabas Hospital Health System, Bronx, USA

**Keywords:** breast lump, hispanic, granuloma, idiopathic, mastitis

## Abstract

Idiopathic granulomatous mastitis (IGM) is a rare benign pathology of inflammation in the breast that commonly affects parous women of reproductive age and men although it is extremely rare. It has an unusual predilection for Hispanic women born outside of the United States, most notably in Mexico. Recently, this entity has been described more. However, the approach to management is still very controversial, and the approaches vary widely, although surgical approaches, including excision or mastectomy, have been less favored recently as a primary approach. Here, we present a case of a young female of reproductive age who presented initially with a suspicious breast lump diagnosed initially in the breast clinic as IGM and was referred to the rheumatology clinic for management with medical therapy.

## Introduction

Idiopathic granulomatous mastitis (IGM) is an inflammatory breast disease with an estimated incidence of 2.4 per 100,000 women and 0.37% in the United States [1], producing a clinical picture that mimics malignancy or an infectious process. However, the etiology has been difficult to identify clearly. Different hypotheses have been proposed, including autoimmune, hormonal, or local immunological reactions to milk leakage from the lobule [2]. Other disease-related factors include elevated prolactin levels, oral contraceptives, pregnancy, and breastfeeding. While the treatment approaches vary depending on the clinical context, surgical approaches have been described, from simple drainage and irrigation to mastectomy. Here, we report a case referred to the rheumatology clinic and managed with medical therapy.

## Case presentation

The patient is a 33-year-old Hispanic female born in Mexico who initially presented to the emergency department (ED) with a left-sided breast lump. She was evaluated and discharged with outpatient follow-up but failed to follow up. She subsequently presented to the breast clinic with a larger mass on the left breast. She had no significant past medical history or family history of breast cancer. The patient started menstruation at the age of 9 and was pregnant once with the outcome of miscarriage at age 28.

**Figure 1:**
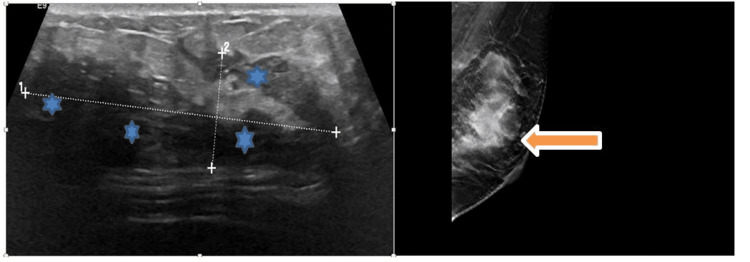
Left: Irregular left breast mass measuring 5.5 x 1.7 cm observed on ultrasound (indicated by stars). Right: mammography showing the same lesion (orange arrow).

On physical examination, the patient was found to have a tender irregular hard mass measuring 8 x 5 x 3 cm in the upper outer quadrant of the left breast without skin changes, nipple inversion, or lymphadenopathy. Mammography revealed a large mass in the central upper breast extending into the axillary tail region of the left breast and two prominent asymmetric appearing left axillary lymph nodes. Ultrasound (US) confirmed the presence of a mass in the left breast (Figure 1). A biopsy was subsequently performed on the affected breast.

**Figure 2:**
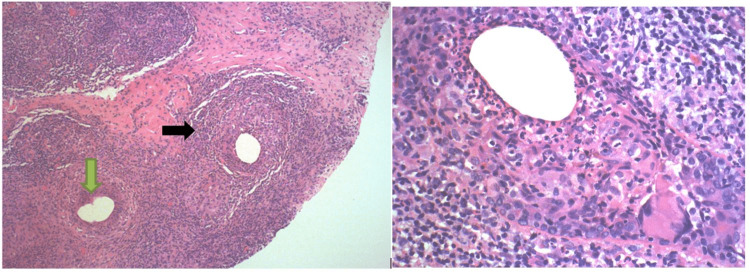
Breast core biopsy showing cystic spaces (green arrow) rimmed by neutrophils and giant cells (black arrow), H&E stain.

The initial core needle biopsy of the left breast mass showed breast tissue with dense mixed acute and chronic inflammation, multinucleated giant cells, and poorly formed granulomas, consistent with IGM. However, a second biopsy was performed on the left breast mass guided by US, which showed breast parenchyma with extensive acute and chronic granulomatous inflammation involving stroma and lobules focally with cystic spaces surrounded by neutrophils and granulomatous inflammation, histologic changes typical of cystic neutrophilic granulomatous mastitis (CNGM) (Figure 2). The biopsy also showed no organisms with acid-fast histochemical and immunohistochemical stains, gram stains, or Gomori methenamine silver stains. While the lymph nodes only showed fibro-adipose tissue with chronic inflammation. 

At the time of the second biopsy, an infectious process was on the differential as the site of the previous biopsy was draining yellowish mucopurulent discharge, and cultures were taken. Then, purulent discharge appeared from the biopsy and nipple sites. The patient received a seven-day treatment of amoxicillin/clavulanic acid while awaiting the findings of the final culture, which showed susceptible *Staphylococcus simulans *on the first culture and no growth on the second culture, which was considered a contaminant.

After completing the seven-day course of antibiotics, she returned to the ED with continued pain and swelling of the left breast. The patient was evaluated by the surgery team, who determined that surgical intervention was not necessary in her case. The ED admitted her for a follow-up of the breast pain. She was started on a 40 mg prednisone regimen and was discharged with notable improvement, including decreased erythema, swelling, no drainage, and decreased tenderness and pain. Laboratory results showed a mildly elevated white blood cell count of 11.0 × 10^3^/μl, with normal inflammatory markers. In order to rule out any possibility of lung sarcoidosis during the hospitalization, a computed tomography (CT) of the chest was also performed, although it did not identify any suspicious lesions. 

The patient experienced hives as a reaction to the outpatient medications prescribed for prednisone therapy. Rheumatology was consulted in response to this, and the dosage of prednisone was adjusted. The patient has gradually weaned off prednisone over three months, and azathioprine was started after one month of prednisone therapy alongside testing for thiopurine methyltransferase, which was revealed to be heterogeneous. The patient reported improvement in the symptoms. Due to heterogeneity and the supporting evidence of methotrexate (MTX), a plan was to switch the patient to MTX, doxycycline, and colchicine after two more months of azathioprine.

**Figure 3:**
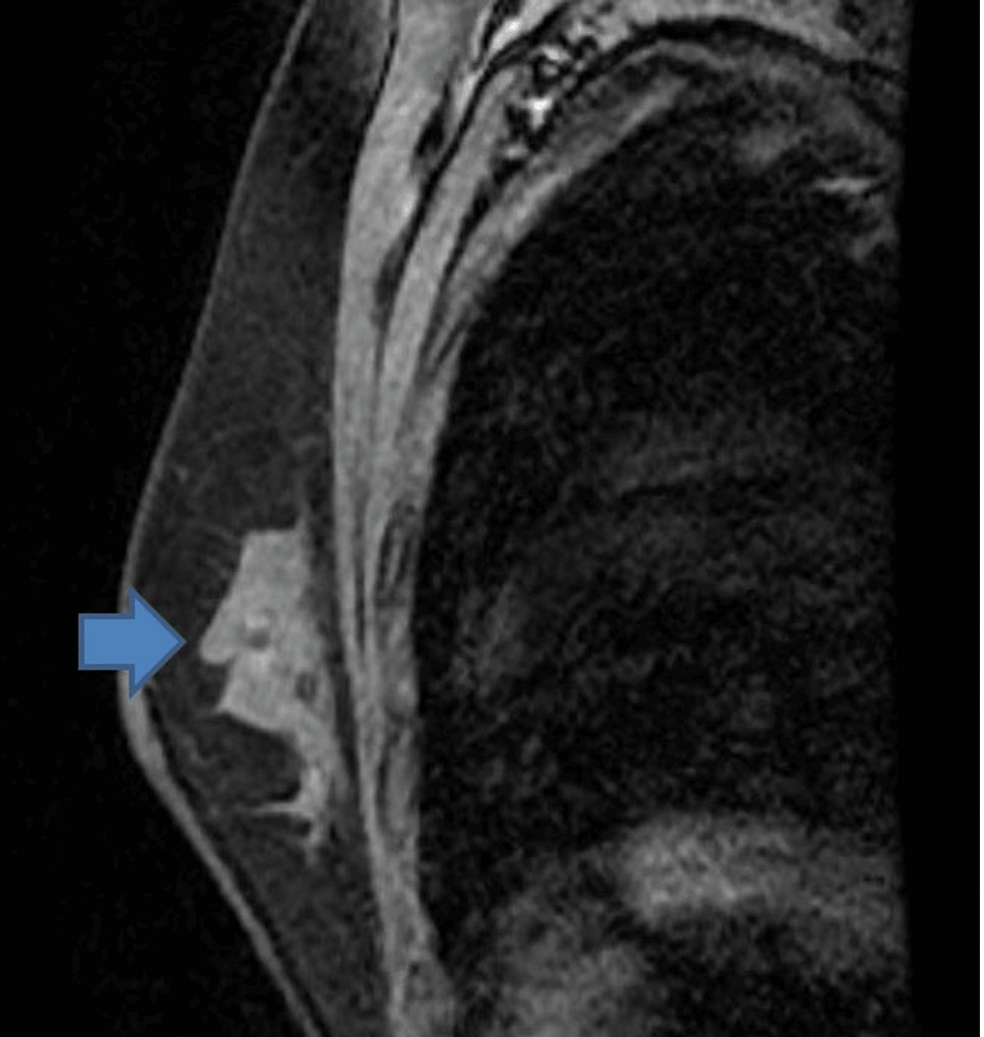
Follow-up MRI showing an irregular left breast mass (blue arrow).

Approximately one year after starting MTX therapy, US and mammogram were performed, which revealed almost complete resolution. On the other hand, a follow-up magnetic resonance imaging (MRI) (Figure 3) showed abnormal enhancement in the left central dominantly outer breast with mild interval improvement. The mass size had decreased from 7.6 x 3.3 cm to 5.4 x 3.6 cm. There was also an interval of mild improvement of the previously noted overlying skin thickening/edema.

The patient returned for follow-up, reporting improvement in symptoms. Colchicine and doxycycline were stopped after 12 and 14 months, respectively. The MTX dose (20 mg weekly and now on 10 mg weekly) was tapered down on her last visit up until the date of this article (total treatment duration of 18 months), reporting resolution of the previous symptoms with no new complaints.

## Discussion

IGM is an exceptionally rare benign illness of breast inflammation that most frequently affects parous women of reproductive age [3,4]. According to numerous studies [1,5,6], it has an uncommon preference for Hispanic women born outside of the United States, particularly those from Mexico. Also, this disease has been discussed more lately [5].

With the disease being increasingly reported, there needs to be a more comprehensive discussion in the rheumatology literature. The nature of the disease and the increasing referrals from gynecologists and breast surgeons make it necessary to be familiar with the course and management of this disease. 

Typically, the most common clinical presentation of IGM is a unilateral palpable painful breast lump. Moreover, it commonly appears with abscess development and moderate cutaneous erythema, areolar retraction, fistula, and ulceration. It is crucial to understand that, given the clinical picture it presents, it can easily be confused with an abscess or an inflammatory form of breast cancer. IGM is an exclusionary diagnosis that can only be made after significant etiologies have been ruled out, where imaging modalities are utilized in the case of identification of a lump with US, mammography, and MRI, followed by a core biopsy of the lesion. 

Histopathology is commonly used to make the final diagnosis of IGM, and this process frequently involves inflammatory cells with noncaseating granulomas. Of notice, the pathology of our patient revealed a "subtype" of this condition known as CNGM, which can be distinguished by the presence of empty "cystic" spaces consistent with dissolved lipid and the identification of the lipophilic bacterium *Corynebacterium kroppenstedtii*, which is most frequently seen on gram stain [7].

Methods of management can be largely divided into two categories: medical and surgical. These approaches differ particularly in the absence of defined guidelines.

Observation

A significant retrospective study conducted in 2019 [8] involved 120 female patients diagnosed with IGM. These patients were managed through monitoring and supportive care, with surgical intervention limited to core biopsy and drainage of fluid collections. The study reported an average time to resolution, defined as the absence of a palpable mass and a residual wound, of five months, with a range of 0 to 20 months. The authors of the study emphasized the importance of educating patients about the typical course of the disease, which includes episodes of exacerbation and recurrence in 16% of the sample before ultimately resolving. This approach is further supported by additional investigations [9,10].

Antibiotics

Antibiotics are usually prescribed to patients if there is an initial suspicion of bacterial infection, with antibiotics targeting the most common pathogens (*Staphylococcus* or *Streptococcus*) such as amoxicillin/clavulanic acid; moreover, it is recommended to treat with lipophilic antibiotics such as doxycycline, clarithromycin, and rifampicin, especially in the CNGM type, where Corynebacterium is identified. However, the role of Corynebacterium as the underlying cause of CNGM remains controversial, as some consider it to be a contaminant from the skin flora [11]. It should be noted that antibiotics alone have limited efficacy in the treatment of this condition, given its complex nature.

Oral, topical, and local injections of steroids

Oral systemic steroids are frequently employed in the treatment of IGM and have shown a success rate of up to 71% [12]. Complete remission of symptoms is typically achieved within a period of 5 to 10 months [13]. However, the use of systemic steroids carries the risk of severe side effects, including myopathy, hyperglycemia, and gastritis, among others. Prednisone is the most commonly prescribed medication for IGM, with doses ranging from 10 to 60 mg/day.

On the other hand, topical steroids were also used in some studies with low recurrence rates ranging from 10 to 14% [13,14]. Furthermore, the agent used in the previous studies was prednisolone ointment (0.125%) twice a day for one to three months.

Studies also noted the use of local steroid injections. A common medication is methylprednisone, which is injected into the breast parenchyma once a month in doses ranging from 40 to 440 mg [15]. Toktas et al. [16] classified 78 female GLM patients into two therapy groups: Group 1 received oral systemic steroid therapy, whereas group 2 received intralesional steroid injection along with topical steroid administration (group 2). While the rates of complications were comparable between the groups, group 2 had much lower rates of recurrence (8.7%) than group 1 (46.9%, P = 0.001), and group 2 required significantly less surgical intervention (2.2% vs. 9.4%, P = 0.001).

Immunosuppressive agents

The most commonly reported agent of choice is MTX. Most of the literature discussing this agent originates from Rheumatology articles; MTX acts as a steroid-sparing agent to reduce the side effects of systemic steroids. In one study, remission rates of 80% were reported, surpassing the rates achieved with steroids alone or a combination of steroids and surgery [17]. Additionally, MTX has demonstrated excellent results in patients who did not achieve remission with steroids or surgery [18,19].

Other medications

it is worth mentioning that colchicine has been reported to be used, as in the case of our patient, with some success; on the other hand, bromocriptine has also been used in severe cases, especially when prolactin levels are elevated, implicating a role for prolactin in the pathophysiology of IGM, with a screening of an adenoma as suggested by some authors [20].

Surgery

Surgery usually achieves a high remission rate, but not without complications, such as infection, bleeding, wound dehiscence, nerve damage, lymphedema, and general complication of any surgery such as increased rate of venous thromboembolism, not to mention the associated anxiety for the patient. Surgical intervention is usually prescribed for patients with a high recurrence rate, abscess formation, and sinus tract formation.

## Conclusions

IGM and its subtype, CNGM, are rare benign breast diseases that can present with a palpable mass in the breast. They can mimic the clinical and radiological features of breast cancer. A high index of suspicion is required for the diagnosis, and a biopsy is essential to exclude malignancy. Treatment options include steroids, immunosuppressants, and surgery. Regular follow-up is required to monitor for recurrence. Physicians should consider the possibility of bilateral involvement in cases where the patient presents with bilateral symptoms or has a history of autoimmune disorders or smoking. In cases where an infectious process is suspected, appropriate cultures should be obtained, and antibiotics may be indicated pending culture results. The development of nipple discharge and discharge from biopsy sites should be carefully monitored and managed accordingly. Moreover, patient education should be a priority for the clinician. 
